# Relationships between the Gut Microbiota of Juvenile Black Sea Bream (*Acanthopagrus schlegelii*) and Associated Environment Compartments in Different Habitats

**DOI:** 10.3390/microorganisms9122557

**Published:** 2021-12-10

**Authors:** Peng Sun, Hui Zhang, Yazhou Jiang, Quanxin Gao, Baojun Tang, Jianzhong Ling, Xingwei Yuan

**Affiliations:** 1 Key Laboratory of East China Sea Fishery Resources Exploitation, Ministry of Agriculture, East China Sea Fisheries Research Institute, Chinese Academy of Fishery Sciences, Shanghai 200090, China; sunpeng0512@hotmail.com (P.S.); zhhui269@126.com (H.Z.); bjtang@yeah.net (B.T.); lingjianzhong18@sina.com (J.L.); xwyuanhust@sina.com (X.Y.); 2 Zhejiang Provincial Key Laboratory of Aquatic Resources Conservation and Development, College of Life Science, Huzhou University, Huzhou 313000, China; gaoqx2008@163.com

**Keywords:** *Acanthopagrus schlegelii*, gut microbiota, diet, habitat, environment

## Abstract

The fish-gut microbiota play a key role in the physiology, development, and fitness of its host. An understanding of fish-gut microbial communities and the factors influencing community composition is crucial for improving fish performance. In this study, we compared the gut microbiota of juvenile black sea bream *Acanthopagrus schlegelii* among habitats: (1) wild, (2) offshore cage-culture, and (3) pond-culture. We also explored the relationships between the gut microbiota and host-associated environmental factors. Gut samples and associated environmental compartments were investigated using 16S rRNA gene sequencing. Our results revealed significant habitat-specific differences among the gut microbiota of juvenile *A. schlegelii*. Wild populations of juvenile *A. schlegelii* had more diverse gut microbiota than populations cultured in pond habitats due to their omnivorous feeding habits and the corresponding abundance of natural food resources. Significant variations in the composition, core taxa, and diversity of the microbiota were also found between the gut and the environmental compartments. However, no significant differences were observed among the microbiota of the environmental compartments in the relatively isolated pond habitat. Source tracking analysis recovered connections between the fish-gut microbiota and the diet, water and sediment environmental compartments. This connection was especially strong between the microbiota of the fish gut and that of the diet in the pond habitat: the diet microbiota accounted for 33.48 ± 0.21% of the gut microbiota. Results suggested that all *A. schlegelii* shared a core gut microbiota, regardless of differences in diet and habitat. However, environmental factors associated with both diet and habitat contributed to the significant differences between the gut microbiota of fish living in different habitats. To the authors’ knowledge, this study presents the first comparison of gut microbiota among juvenile *A. schlegelii* with different diets and habitats. These findings enrich our understanding of the gut microbiota of *A. schlegelii* and help to clarify the interaction between gut microbiota and environmental factors. Our results may also help to guide and improve fish ecological fitness via the regulation of gut microbiota, thereby increasing the efficacy of stock enhancement programs for this species.

## 1. Introduction

The black sea bream (*Acanthopagrus schlegelii*), a commercially important marine fish species, is widely distribution in the estuaries and embayments of China, Japan, and Korea. Due to intense fishing pressure, the catch of this species has drastically declined, and stock enhancement programs were first enacted in the 1980s to restore depleted populations of *A. schlegelii* on the southeastern coasts of China and Japan [1,2]. Because hatchery-released fish populations often have relatively low post-release success rates in terms of survival, behavior, or breeding performance [3], short-term post-release survival is viewed as a crucial first step for the success of stock enhancement programs [4].

The gut microbiota have been shown to affect the metabolic capacity of the fish host and to have beneficial effects on host nutrition, growth, reproduction, and health [5,6,7,8,9,10,11]. Hence, studies have increasingly focused on exploring the fish-gut microbiome, with the long-term goal of developing healthy and sustainable aquaculture production systems [12]. A recent study showed that diet training using the natural diet improved the reintroduction success of *Acipenser dabryanus* by adjusting the pre-release gut microbial community [3]. The structure, composition, and ecological functions of the fish-gut microbiota can be influenced by a wide range of intrinsic and extrinsic factors, including host genetic background, physiology, diet, living environment, antibiotic utilization, and probiotic utilization, as well as farm management practices [13,14,15,16]. However, the interactions between the environmental microbiota and the gut microbiota in cultured fish species have received insufficient attention [11], especially in juvenile populations used for stock enhancement purposes in species such as *A. schlegelii*.

Hatchery-released populations of *A. schlegelii* are usually reared in offshore cages or in ponds and are fed commercial feed or raw fish, both of which differ markedly from the diets and habitats of the corresponding wild populations [1,2]. If released populations cannot rapidly adapt to the conditions in the wild environment, the physiological development and health of these fish will be affected. Therefore, to clarify these interactions, we investigated the gut microbiota of juvenile *A. schlegelii* in different habitats, as well as microbiotal differences associated with different dietary and environmental compartments (e.g., water and sediment), using high-throughput sequencing of the 16S rRNA gene. We aimed (1) to characterize the composition and diversity of the microbiota in the gut of juvenile *A. schlegelii* and the associated environmental compartments; and (2) to determine the differences in gut microbiota among diet and habitats, exploring connections between the gut microbiota and host-associated environmental factors. This study may provide insights into the composition, diversity, and function of the gut microbiota of *A. schlegelii,* and may also help to clarify how the microbiota interact with diets and factors associated with habitat in this species. Our findings may also contribute to the future development of improved aquaculture systems for *A. schlegelii* that improve efficacy, fish health, ecological fitness, and the stock enhancement.

## 2. Materials and Methods

### 2.1. Sample Collection and Grouping

The *A. schlegelii* used in this study (collected from 19 to 23 July 2020) can be grouped into three types based on habitat: (1) wild-caught juveniles from Xiangshan Bay (China; 29°30′15″ N, 121°34′14″ E to 29°30′30″ N, 121°35′50″ E) using brief small-scale trawling (group WG), (2) cage-cultured populations in the Xiangshan Bay area (29°27′36″ N, 121°32′27″ E), reared for release operations (group CG), and (3) seawater pond-cultured populations from Ninghai County (China; 29°8′44″ N, 121°30′53″ E), reared for release operations (group PG). Xiangshan Bay, a semi-closed bay on the central coast of Zhejiang province, China, is an important breeding ground for many economically important species and plays an important role in maintaining the sustainable development of the coastal fishery resources in the East China Sea. The ponds used in this study were located in Ninghai County, Zhejiang province, China. Ponds were approximately 2.4 m deep and were naturally lit. The ponds were managed daily. Individual fish were randomly selected, caught with a net, and euthanized with an overdose of neutralized MS 222. After routine biological measurements, including body length and body weight, 27 individuals (9 individuals per group), with an average body length of 60.65 ± 1.23 mm and an average body weight of 2.03 ± 0.34 g (mean ± S.E.), were dissected under sterile conditions. The complete gut tissues were collected into sterile Eppendorf tubes and stored at −80 °C.

To better understand the environmental factors that influence the fish-gut microbiota, we collected samples of the pellet feed fed to the caged (CD) and pond-cultured (PD) fish. Environmental samples (e.g., water and sediment) were also collected, although because the cages were kept in the deep seawater area, only sediment samples from the ponds (PS) were collected. These samples were divided into nine groups (sample information is shown in Table S1). Sediment samples from each of the three sites were collected using a Petersen grab. Samples collected from three locations at each site (3 g per location) were mixed, and 100 mg of the pooled sample was used for DNA extraction. The surface water in the natural sea area (WW, 5 sites), the cage-culture area (CW, 5 sites) and the pond (PW, only 3 sites because the water area was so much smaller than the other sites) were sampled using a water collector. At each site (13 sites in total), 100 mL of water was collected from each of six randomly selected locations, and pooled (600 mL in total for each site) into one sample. Each water sample (13 samples in total, with distilled water as the control) was filtered through a GF/C member with pore size of 0.22 μm (Whatman, Maidstone, UK). The used filter membranes were cut into pieces for DNA extraction; blank membranes were used as controls.

### 2.2. Measurement of Physicochemical Aquatic Properties

Water temperature, salinity, PH, and dissolved oxygen were measured in the field using a Sea-Bird SBE 37 CTD (Sea-Bird Scientific, Bellevue, WA, USA) and a smart portable multiparameter water quality analyzer (YSI 6600; Xylem Analytics, Rye Brook, NY, USA). Water transparency, ammonium nitrogen (NH_4_^+^-N), nitrate nitrogen (NO_3_^−^-N), nitrite nitrogen (NO_2_^−^-N), total nitrogen, and total phosphorus were determined following standard methods [17].

### 2.3. Genomic DNA Extraction and 16S rRNA Sequencing

The 49 samples (27 fish tissue samples, 13 water samples, 6 feed samples, and 3 sediment samples) were collected in sterile centrifuge tubes, and total genomic DNA was extracted using bacterial DNA extraction kits (Tiangen, Beijing, China), following the manufacturer’s instructions. Extracted DNA was quantified by NanoDrop ND-2000 spetrophotometer (Thermo scientific, Waltham, WA, USA) and then visualized on 1.0% agarose gels for quality and integrity verification; DNA extracts were stored at −20°C until further analysis were performed.

From each extracted DNA sample, a fragment of the 16S rRNA V3–V4 region was amplified using the primer pair 338F (5′-ACTCCTACGGGAGGCAGCA-3′) and 806R (5′-GGACTACHVGGGTWTCTAAT-3′) [18]. PCR amplification was performed in a total volume of 25 μL, which contained 5 μL of 5× reaction buffer, 5 μL of 5× high GC buffer, 2 μL of dNTPs (10 mM), 2 μL template DNA, 1 μL 10 μM of each primer, and 0.25 μL of Q5 high-fidelity DNA polymerase (NEB, Ipswich, MA, USA). After an initial denaturation at 95 °C for 3 min, the reactions were run for 30 thermal cycles with denaturation at 95 °C for 30 s, annealing at 50 °C for 30 s and extension at 72 °C for 30 s, followed by a final extension at 72 °C for 5 min. The processed PCR products were sent to Personalbio Biotechnology Co., Ltd. (Shanghai, China) for paired-end sequencing. High-throughput sequencing was performed using an Illumina Novaseq platform.

### 2.4. Data Analysis

The raw sequences obtained from the Illumina Novaseq platform were initially screened and divided. Then, the barcode sequence was removed. We used QIIME2 dada2 [19] to quality filter, denoise, dereplicate and perform a final cluster of the sequences into amplicon sequence variants (ASVs) with 100% similarity. The processed and ASV-grouped sequences were annotated against the Silva database (http://www.arb-silva.de/, accessed date 29 August 2020) [20] using the classify-sklearn algorithm in QIIME2 [21]. The ASVs shared and unique among groups were represented by a scale-Venn diagram, which was drawn using the VennDiagram package. Based on the ASV statistics, the specific composition of the microbial community in each sample at each classification level was obtained. A circle packing chart was drawn to show the taxonomic composition of the microbial community using the R software.

The alpha diversity of each microbiota was assessed using QIIME2; chao1 and observed species were used to estimate species richness, while the Shannon and Simpson indexes were used to estimate diversity. A heatmap of the samples, which were clustered based on Bray-Curtis distances, was drawn using R. Linear discriminant analysis effect size (LEfSe) was determined using R to identify the significantly enriched species in each group. To visualize differences among groups of samples, the Bray-Curtis distance matrix was also used to generate a principal coordinate analysis (PCoA) plot using QIIME2. In addition, a nonmetric multidimensional scaling (NMDS) analysis was performed in R [22] to depict sample distributions. NMDS analysis only consider relative, not absolute, differences in inter-sample distances, and it is generally believed that stress values of less than 0.2 indicate more reliable NMDS results [23]. To test the effects of various explanatory variables (i.e., habitat, diet, and the environmental compartments of water and sediment) on the groupings of microbial communities, analysis of similarities (ANOSIMs) in the distance matrix were performed in QIIME2, and significance was determined based on 10,000 permutations [16,24]. Source tracking analysis was conducted using the Source Tracker package in R [25]. To predict the metabolic activity of the gut microbiota of different groups, Phylogenetic Investigation of Communities by Reconstruction of Unobserved States (PICRUSt) was used to construct the Kyoto Encyclopedia of Genes and Genomes (KEGG) pathway in PICRUSt2 software. Based on 16S information and key findings of the Human Microbiome Project, PICRUSt can be used to accurately predict the abundance of gene families in host-associated and environmental communities [26]. Data are presented as the mean ± standard error for each group; *p*-values < 0.05 were considered to be statistically significant.

## 3. Results

### 3.1. Aquatic Physicochemical Factors in Different Habitats

The main water quality parameters of the three habitats of the sampled juvenile *A. schlegelii* are shown in Table 1. For the two marine habitats, temperature, PH, and salinity were similar. Water transparency and dissolved oxygen was lower in the cage-culture area as compared to wild natural sea area, but no other significant differences in aquatic physicochemical factors were found between the two marine habitats (*p* > 0.05). Compared to the marine habitats, the pond habitat had lower levels of salinity, transparency and dissolved oxygen, and significantly higher levels of ammonium nitrogen, nitrate nitrogen, nitrite nitrogen, total nitrogen, and total phosphorus (*p* < 0.05).

### 3.2. Sequencing Data and Diversity Analysis

**Figure 1:**
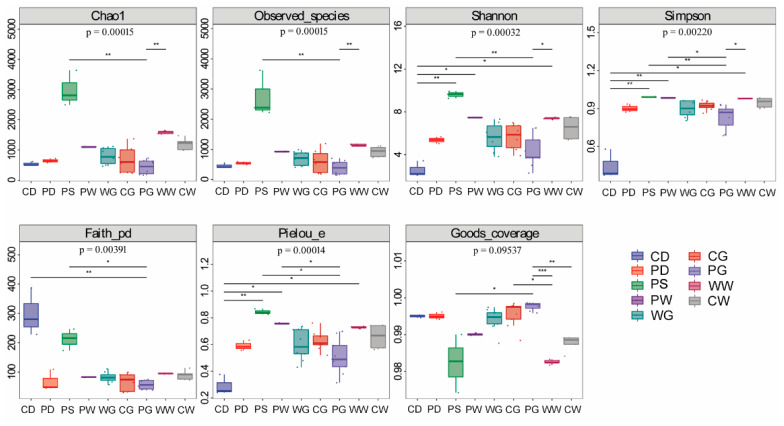
Alpha diversity of the microbiota associated with fish gut, diet, and environmental compartments. Boxplots of microbial community from different groups are outlined in different color; boxes cover the interquartile range (IQR) and the line inside the box denotes the median. Whiskers represent the lowest and highest values within 1.5× IQR. CD, diet for cage-cultured fish; CG, gut of cage-cultured fish; CW, water from cage culture area; PD, diet for pond-cultured fish; PG, gut of pond-cultured fish; PS, sediment from pond; PW, water from pond; WG, gut of wild-caught fish; WW, water from natural sea area. Asterisk indicated significant difference between two groups, among them, * indicates *p* < 0.05, **, indicates *p* < 0.01, ***, indicates *p* < 0.001.

A total of 3,770,729 high-quality sequences were obtained across the 49 samples. After dereplication, 30,117 ASVs were obtained and were used in subsequent analyses. To fully assess the alpha diversity of the microbial community, seven diversity indices were calculated in this study. Of these, chao1 and observed species were used to estimate species richness, the Shannon and Simpson indexes were used to estimate diversity, Faith’s PD was used to estimate phylogenetic diversity, Pielou’s evenness was used to estimate microbial community evenness, and Good’s coverage was used to estimate species coverage (Figure 1). The Good’s coverage estimate was 99.24 ± 0.01% for all of the samples, indicating that the majority of the microbial species had been detected. Alpha diversity indices differed significantly among the gut-, diet-, water-, and sediment-associated microbiota. The microbiota of the sediment from the pond habitat (PS) had the greatest species abundance (chao1, 2980.31 ± 0.33; observed species richness, 2743.57 ± 763.05) and diversity (Shannon index, 9.61 ± 589.37; Simpson index, 0.99 ± 0.00), followed by the microbiota of the water samples from all three habitats. The microbiota of the PG and PD groups had the lowest species abundance (chao1, 431.50 ± 222.40; observed species richness, 394.01 ± 222.41) and diversity (Shannon index, 3.96 ± 1.61; Simpson index, 0.67 ± 0.26).

In addition, significant differences in diversity were found among the microbiota of the gut, diet, and environmental compartments in the pond habitat (Figure 1). *A. schlegelii* from the natural sea area (WG) had the most abundant microbiota, followed by fish from cage-cultured habitat (CG). Conversely, fish in CG group had the most diverse microbiota, followed by the WG group. However, there were no significant differences in either abundance or diversity among the three habitats.

### 3.3. Taxonomic Composition of Microbiota Associated with Gut, Diet, and Environmental Compartments

Rarefaction curves of all groups reached saturation plateaus, indicating that the sequencing depth of all samples was sufficient to accurately reflect microbial community structure and diversity (Figure S1). A multi-Venn diagram (Figure S2) was used to identify the ASVs shared and unique among groups. Gut and water samples from the natural sea area had the most unique ASVs, while gut and water samples from the pond had the least. In addition, 8.70% of the ASVs were shared between the WG and CG groups, while 6.41% were shared between the WG and PG groups. In total, 3.03% of the ASVs were shared across all gut samples, and 0.46% of the ASVs were shared across all of the water samples. Finally, the percentages of ASVs shared between the gut and the environment in the wild, cage-cultured, and pond-cultured groups were 3.10%, 7.01%, and 7.65%, respectively.

We identified 61 phyla across the gut, diet, and environment compartments: three archaeal phyla and 58 bacterial phyla. The five dominant phyla were Proteobacteria, Cyanobacteria, Firmicutes, Actinobacteria and Bacteroidetes. These phyla comprised 94.11 ± 1.76% of the analyzed high-quality sequences. The mean relative abundances of the 10 most abundant phyla and families in the microbiota of each group are shown in Figure S3. The phylum Cyanobacteria was predominant in the CD, PD, and PW groups, while Firmicutes was the most abundant phylum in the CW group. The most abundant phylum in the gut, PS, and WW groups was Proteobacteria. The most dominant family was Synechococcaceae, followed by Moraxellaceae, Comamonadaceae, Planococcaceae, and Micrococcaceae. In particular, Synechococcaceae was the most abundant family in the PM, PW, and WW groups, and Moraxellaceae was predominant in the CG group. Comamonadaceae and Planococcaceae were the most abundant phyla in the CD and CW groups, respectively.

**Figure 2:**
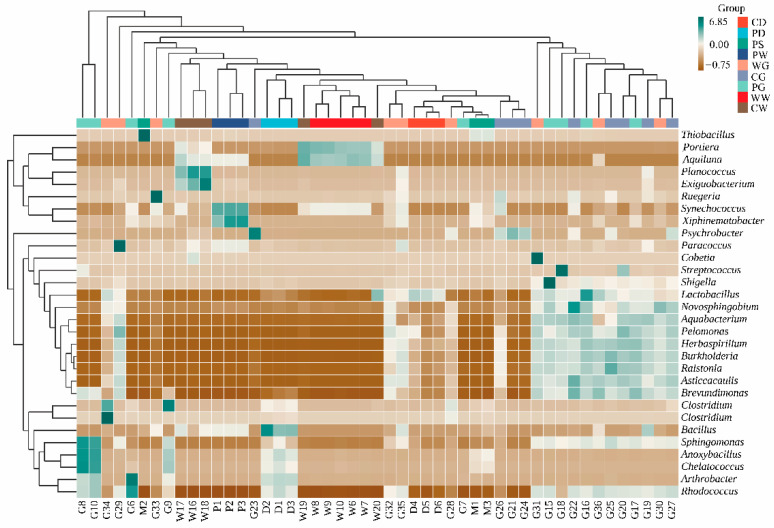
Heatmap showing genus-level microbial composition. The relative abundance shown represents the mean relative abundance for the corresponding group.

To evaluate microbiotal differences between samples and to visualize trends in the distributions of microbial abundance among groups, we used a heatmap to further compare the 30 most abundant genera (Figure 2). Cluster analysis showed distinct patterns of microbiota compositions among the gut, diet, water, and sediment microbiota. Also, differences among groups of gut microbiota were less marked when compared with the microbiota of the associated environmental compartments.

### 3.4. Habitat-Associated Differences in the Gut Microbiota of Juvenile A. schlegelii

The five most abundant phyla in the guts of *A. schlegelii* were Proteobacteria, Firmicutes, Actinobacteria, Cyanobacteria, and Bacteroidetes. Across all habitats investigated, the gut microbiota were dominated by Proteobacteria and Firmicutes, and all gut microbiota contained a similar relative abundance of Bacteroidetes. However, compared with the WG group, the PG group had a greater relative abundance of Actinobacteria and a lower relative abundance of Cyanobacteria, while the CG group had lower relative abundances of Firmicutes, Actinobacteria, and Cyanobacteria.

*Aquabacterium* and *Burkholderia* were dominant genera in the gut microbiota across all three habitats. A LEfSe cladogram was used to characterize the gut microbiota of *A. schlegelii*, and significant differences in abundance were observed among groups living in different habitats (Figure S4). Consistent with the diversity analysis, the PG group had fewer characteristic taxa (i.e., taxa that were significantly more abundant in the PG group as compared to the WG and CG groups). The Phyla Firmicutes and Chlorobi were significantly more abundant in the WG group, while Proteobacteria was significantly more abundant in the CG group as compared to the WG and PG groups. Across the genera identified, *Bacillus*, *Anaerospora*, *Labrenzia*, and *Ruegeria* were found in both the WG and CG groups, *Anoxybacillus* was found in both the WG and PG groups, and *Chelatococcus* and *Methylotenera* were found in both the CG and PG groups. In contrast, *Microbulbifer*, *Cronobacter*, *Acinetobacter*, and *Psychrobacter* were only abundant in the CG group, while *Arthrobacter* was only abundant in the PG group. PICRUSt analysis indicated that the gut microbiota of *A. schlegelii* exhibited similar patterns of gene expression across groups, including genes associated with amino acid biosynthesis, cofactors, prosthetic group metabolic processes, electron carriers, vitamin biosynthesis, nucleoside and nucleotide biosynthesis, fatty acid and lipid biosynthesis, carbohydrate biosynthesis, cell structure biosynthesis, fermentation, and the tricarboxylic acid (TCA) cycle. However, genes in the starch biosynthesis pathway were significantly downregulated in the PG group as compared to the WG group (log2FC = 1.322, *p* < 0.01).

### 3.5. Connections among the Microbiota of the Gut, Diet, and Other Environmental Compartments

**Figure 3:**
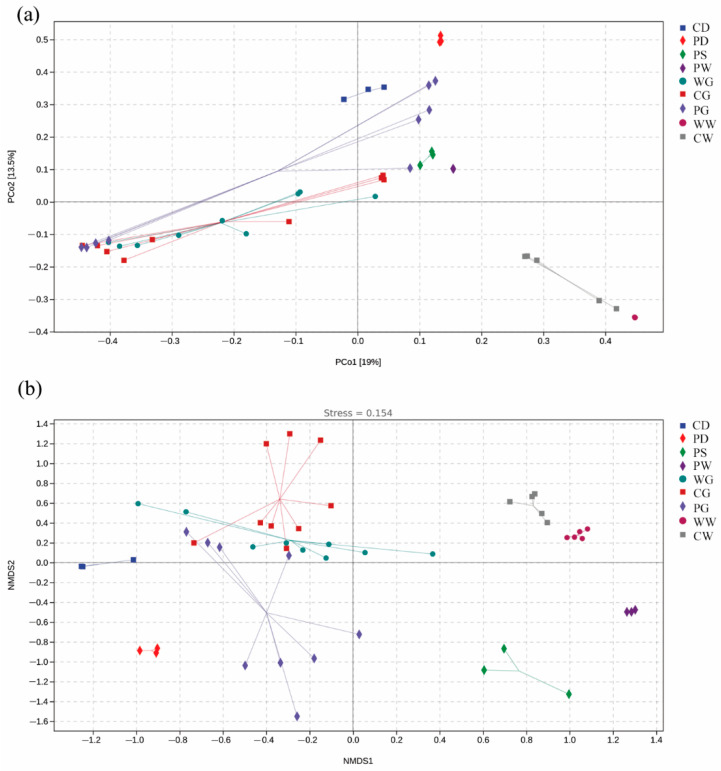
Distributions of the microbial communities of the different groups. (**a**) principal coordinates analysis (PCoA); (**b**) nonmetric multidimensional scaling (NMDS) analysis.

The PCoA and NMDS analyses showed that the diet, water, and sediment groups formed distinct clusters that were separated from the fish gut groups, whereas the gut groups generally clustered together (Figure 3). ANOSIM also revealed significant differences both among the gut microbiota of fish living in different habitats (*p* < 0.01), and between the gut microbiota and the microbiota of the environmental compartments (*p* < 0.05; Table S2).

**Figure 4:**
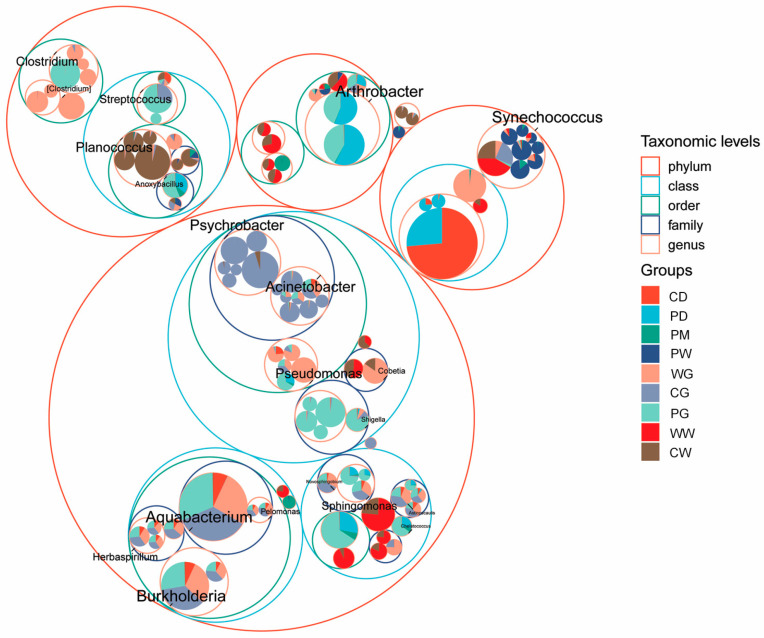
Taxonomic tree showing the microbial community associated with *A. schlegelii* gut, diet, and environmental compartments in different living habitats. The largest circles represent the phylum level, and the inner circles represent class, family, and genus. The circle sizes represent the mean relative abundance of the differentially abundant taxa. The sector area within a circle indicates the abundance of the taxon (top 100) to which the circle corresponds.

However, analyses showed that certain microbes were shared among groups, indicating connections between the gut microbiota and those from other sources. That is, a circle packing chart of the genus-level microbial communities of the gut, diet, and environmental compartments (Figure 4) showed that *Synechococcus* was found in all of the gut, sediment, and water samples; *Arthrobacter* dominated the PD and PG groups, but was also found with low relative abundances in the PS and CD groups; *Planococcus* dominated the CW group, but was also found in the WG and CG groups; *Psychrobacter* dominated the CG group, but was also found in the WG and CW groups; and *Anoxybacillus* and *Chelatococcus* were found together in the PG, PD, and PS groups. Several species were found only in specific groups: *Psychrobacter pacificensis* and *Psychrobacter celer* were only found in the CG and CW groups; *Pseudomonas alcaligenes* was only found in the PG, PD, and PS groups; *Sphingomonas yabuuchiae* dominated the PD and PG groups; *Photobacterium damselae*, *Paracoccus aminovorans*, and *Paracoccus zeaxanthinifaciens* were only found in the WG group; *Psychrobacter pulmonis* and *Lawsonia intracellularis* were only found in the CG group; *Streptococcus agalactiae* was only found in the PG group; and *Pseudomonas fragi*, *Ruegeria lacuscaerulensis*, and *Anaerospora hongkongensis* were predominant in the WG group, but were also found in the CG group with relative abundances.

Source tracking analysis was used to evaluate the contribution of the diet- and habitat-associated microbiota to the fish gut microbiota. Across the three habitats, 2.02 ± 0.23%, 1.49 ± 0.27%, and 0.38 ± 0.07% of the bacteria in the gut microbiota were associated with water (in the WW, CW, and PW groups, respectively). A smaller percentage of the fish gut microbiota was derived from the sediment (0.17 ± 0.11%) in the PS group. Interestingly, significant differences in the contribution to the gut microbiota were identified between the PD (33.48 ± 0.21%) and CD (0.82 ± 0.35%) groups, and between the PD group and each of the three water groups (*p* < 0.05). In the cage-culture habitat there was no significant difference between the proportion of the microbiota derived from water and that derived from diet (*p* > 0.05), while in the pond-culture habitat there was no significant difference between the proportion of the microbiota derived from water and that derived from sediment (*p* > 0.05). However, the contribution of the PD group to the gut microbiota was significantly greater than that of any other group. These results indicated that (1) *A. schlegelii* juveniles obtain more microbes from water than from sediment, and (2) that the gut microbiota of the fish in the pond habitat were more influenced by their diets than the gut microbiota of the cage-cultured fish or than by any other habitat-associated factors.

## 4. Discussion

It is well established that microbiota play essential roles in host nutrition, physiology, and health [6,9]. However, little is known about the composition, diversity, and function of the microbiota associated with different environmental compartments and habitats in juvenile *A. schlegelii*. In this study, we compared the gut microbiota of fish populations among different habitats, aiming to better understand how exogenous and endogenous factors influence the gut microbiota of *A. schlegelii*. We hypothesized that comparisons among fish in different habitats might reveal the influences of diet and habitat on the microbial community in the fish gut. For instance, the WG and CG groups are both found in similar marine areas, and comparisons between these groups might reveal the influence of diet and microhabitat on the gut microbiota. Similarly, comparisons between the WG and PG groups might reveal the influences of pond culture (which differ from wild-caught fish with respect to both diet and other environmental compartments) on the gut microbiota. Finally, comparisons between CG and PG might reveal the influences of different habitats on the gut microbiota when the diet is similar. Our results revealed significant variations in the composition, core taxa, and diversity among the microbiota of the gut, feed, and habitat-associated environmental compartments. In addition, the gut microbiota differed significantly among fish living in different habitats (*p* < 0.01), which suggested a connection between the gut microbiota of the juvenile black sea bream and associated environment compartments in various habitats.

The identification of dominant gut microbes is the basis of gut microbiome studies. The fish gut harbors many complex and dynamic microorganisms, among which bacteria are the dominant group [27]. Studies have shown that Proteobacteria, Firmicutes, Bacteroidetes, Actinobacteria, Fusobacteria, Clostridia, Bacilli, and Verrucomicrobia are the most common gut microbes in fish, among which Proteobacteria, Firmicutes, and Bacteroidetes represent the dominant microbes in the gut microbiota in many fish species [28,29,30,31]. In addition, Huang et al. [32] showed that the gut microbiota of adult *A. schlegelii* consist of Proteobacteria, Fusobacteria, Cyanobacteria, Actinobacteria, Firmicutes, and Bacteroidetes. Here, Proteobacteria, Firmicutes, Actinobacteria, Cyanobacteria, and Bacteroidetes were the most abundant phyla in the gut microbiota of juvenile *A. schlegelii*, which was in general consistent with the results in adults. This was also consistent with our results, and Deng et al. [33] showed that Firmicutes and Proteobateria were highly abundant in guts of cultured *A. schlegelii*; the most abundant classes in Firmicutes were primarily Bacilli and Clostridia, while the most abundant classes in Proteobateria were primarily Gammaproteobacteria and Alphaproteobacteria.

The presence of similar gut microbiota across multiple fish populations representing one or more species with different backgrounds (i.e., a core gut microbiota) reflects the important functions performed by these microbes in the host, which may include digestion, nutrient absorption, and immune responses [34]. Knowledge of the core microbiota is critical for an understanding of the assembly and stability of microbial communities; indeed, shared microbiota may be shaped by evolutionarily conserved aspects of digestive tract anatomy, physiology, and immunity [8]. It has been demonstrated that phylogenetic relationships among hosts underlie variations in fish-gut microbiota [8]. Roeselers et al. [34] showed that a core set of microbial genera were shared among domesticated zebrafish populations and wild-caught zebrafish from different geographic locations. In contrast, the microbiota of fish collected from the same area may be species specific: for example, coral reef fish sharing the same habitat have different microbiota due to variations in diet [7,35,36]. Similarly, carp species cohabiting in the same pond may have different microbiota [37]. The host may affect the microbial community by selecting specialized microbiota for digestion and absorption of nutrients from a variety of food sources [8]. Therefore, the gut microbiota are not a simple reflection of the microbial community in the local habitat, but are rather a result of species-specific selective pressures dependent on physiological characteristics. The composition and balance of microbiota can strongly impact the function of the fish physiological processes. In our study, many taxa in the core microbiota are related to nutrient metabolism and the immune response, including species in the genera *Aquabacterium*, *Burkholderia*, *Herbaspirillum*, *Novosphingobium*, and *Lactobacillus*, which was consistent with results of Roeselers et al. [34]. In addition, a high abundance of Firmicutes was found in the WW group. Firmicutes can control the energy balance in animals [38], hence, an efficient function on energy balance in fish of the WW group was suggested. To predict the metabolic activity of the gut microbiota in different groups, PICRUSt was used to construct a KEGG pathway, and the results indicated that the gut microbiota of juvenile *A. schlegelii* were associated with similar gene functions, including amino acids, nucleotides, fatty acids, carbohydrates, and vitamin biosynthesis, irrespective of group. PICRUSt analysis also revealed a significant decrease of the gene expression level in the starch biosynthesis pathway of the PG group when compared with microbiota in the WG group (logFC = 1.322, *p* < 0.01). Considering the feeding needs of *A. schlegelii* and the abundance of plant sourced food in the WW habitat, this decrease might be related to the lower intake of plant foods (e.g., *Ulva lactica*, *Bryopsis corticulans*, and *U. prolifera*) by fish populations in the pond habitat [39]. Generally, plant feed is one of the main components for juvenile *A. schlegelii*, and inefficient ingestion and digestion of plant food may affect the ecological fitness of these fish to the wild habitat once they were released. However, PICRUSt relies on a database mostly composed of bacterial strains from humans [26], and such functional inference must be taken with caution due to the lack of fish associated bacterial symbionts in the database.

An understanding of the effects of various factors on the gut microbiota adjustments is essential for the improvement of physiological performance in the released populations of juvenile *A. schlegelii*. Diet is an important factor affecting the gut microbiota. It has been supposed that fish with more generalized diets carry more diverse microbiota [16], and studies have shown that fish-gut microbial diversity increases from carnivores to omnivores to herbivores [7,40]. For instance, although *Hypophthalmichthys nobilis* and *H. molitrix* belong to the same genus and exhibit similar feeding behavior, the difference in diet between the two species (phytoplankton and zooplankton, respectively) contributes to the significant difference in composition between their gut microbiota [36]. As expected, the wild population of *A. schlegelii* in this study had more diverse gut microbiota than the pond cultured population. Wild populations are omnivorous and their natural diets are extremely varied, possibly contributing to the higher microbial diversity found in their guts.

Diet has been identified as an important influence on gut microbial diversity and community structure in some fish. For example, a study of gibel carp (*Carassius auratus gibelio*) found that 37.95% of the Operational Taxonomic Units (OTUs) detected in feed were also retrieved in the gut, which suggested that diet may markedly influence the gut microbiota of this species [41]. Here, 7.33–16.78% of ASVs in the feed were also found in the gut microbiota, suggesting that diet influenced the gut microbiota of *A. schlegelii*. For example, the genera *Anoxybacillus* and *Chelatococcus* were found in both the PG and PD groups, while *Arthrobacter* only dominated in the PD and PG groups. This directly reflected the microbial connection between the gut and feed microbiota. The gut microbiota might influence the diet preference of the fish host, which might in turn impact the adaptation of the fish to diet provision transformation, determining the fitness and survival of the post-release fish populations. Yang et al. [3] found that when diets provided were more similar to the diet in the natural environment, the individuals of *A. dabryanus* would have a higher survival rate and better growth characteristics after release. Likewise, hatchery-released populations of *A. schlegelii* are often reared with commercial feed or raw fish, which is quite different from diet of the wild populations. Considering the significant differences in gut microbiota between cultured and wild populations of *A. schlegelii* identified herein, a more diverse microbiota is preferred before release. And the improved pre-release diet training, in conjunction with a more appropriate training diet, might appropriately adjust the pre-release gut microbiota of *A. schlegelii* and thus improve reintroduction success. However, further studies are needed to confirm this possibility.

Because it is in constant contact with the environment, the structure and composition of the fish-gut microbiota may be impacted by the environmental microbiota. The acquisition of microbes from the surrounding environment is considered to be the primary method by which fish obtain microbes, and fish have been shown to selectively ingest microbes for microbiotal enrichment from their living habitats from early development onwards [42]. However, the effects of environmental factors on the fish-gut microbiota also seem to vary by species. For instance, the gut microbiota of the grass carp is notably enriched from the surrounding water and sediment [41], while the gut microbota of *H. nobilis* and *H. molitrix* were more similar to the water microbiota than to the sediment microbiota [36]. Finally, sediment was the most important factor determining the gut microbiota of the gible carp [43]. These differences among fish species may be related to corresponding differences in living habits. For example, gible carp are a demersal fish, which inhabit middle and lower water layers, while *H. nobilis* and *H. molitrix* are filter-feeding fish that inhabit the upper water layer. Source tracking analysis revealed that the water and sediment microbiota made limited contributions to the gut microbiota, and similar results were recovered for the CD feed group. However, the contribution of the PD group to the gut microbiota was significantly greater than that of CD or any other host-associated environmental factors (*p* < 0.05). This was consistent with a previous analysis of gibel carp cultured in an artificial pond [41]. These results suggested that diet may markedly influence the gut microbiota of *A. schlegelii* populations cultured in pond habitats. This relationship may be associated with unique physicochemical conditions, relatively small size, and limited water exchange capacity in the pond habitat, but these causes warrant further study. In summary, we have illustrated herein the possible impacts of diet and habitat-associated environmental factors on the gut microbiota of *A. schlegelii*.

## 5. Conclusions

Our study provided the first characterization of the gut microbiota of juvenile populations of *A. schlegelii* with different diets and habitats. Our work provided a comparative structural analysis of the microbiota of fish guts and host-associated environmental compartments. We found significant variations in the composition, core taxa and diversity among the water-, diet-, sediment-, and gut-associated microbiota. In addition, the diet- and gut-associated microbiota were found to be relatively tightly linked in the pond habitat. The environmental factors of water and sediment also influenced the gut microbiota, irrespective of habitat. These findings improve our understanding of the composition and function of the host microbiota of *A. schlegelii*, as well as the environmental factors that influence the gut microbiota of *A. schlegelii* in different habitats.

## Figures and Tables

**Table 1:** The main water quality parameters in different habitats.

Parameters	Habitat Types		
	Wild	Cage	Pond
Temperature (°C)	32.3	32.2	33.1
PH	8.10	8.09	8.02
Transparency (cm)	95	80	18
Salinity	28.75 ± 0.14	28.98 ± 0.31	22 ± 0.18 *
Dissolved oxygen (mg/L)	5.29 ± 0.08	5.12 ± 0.15	4.10 ± 0.08 *
Ammonium nitrogen (mg/L)	0.02 ± 0.00	0.01 ± 0.00	0.43 ± 0.22 *
Nitrate nitrogen (mg/L)	0.49 ± 0.03	0.49 ± 0.08	3.66 ± 0.58 *
Nitrite nitrogen (mg/L)	0.05 ± 0.01	0.04 ± 0.02	0.18 ± 0.03 *
Total nitrogen (mg/L)	0.55 ± 0.06	0.57 ± 0.03	4.56 ± 0.31 *
Total phosphorus (mg/L)	0.06 ± 0.03	0.05 ± 0.01	0.82 ± 0.13 *

* indicates significant difference among groups.

## Data Availability

The merged DNA sequences were deposited in the Genome Sequence Archive with the accession number CRA005221 (https://ngdc.cncb.ac.cn/gsa/browse/CRA005221) and CRA005222 (https://ngdc.cncb.ac.cn/gsa/browse/CRA005222) accessed on 21 October 2021.

## Supplementary Materials

The following are available online at https://www.mdpi.com/article/10.3390/microorganisms9122557/s1, Figure S1: Rarefaction analysis of the high-throughput sequencing reads in different groups; Figure S2: Venn diagram of shared and unique ASVs for (a) gut, (b) water and (c) diet samples; Figure S3: Relative abundance of microbial community proportions at (a) phylum and (b) family level from gut of *Acanthopagrus schlegelii* and environmental compartments in different living habitats. Figure S4: Cladogram of linear discriminant analysis effect size (LEfSe) for difference analysis of gut microbial community in different groups. Classification of the top 30 taxa are marked with letters (from inner to outer, which indicate classification from phylum to genus, respectively). All taxa are shown as nodes. Hollow nodes represent taxa with no significant inter-group differences, nodes and sectors in other colors indicate significant differences between groups, and node sizes are proportional to the average relative abundance of the taxa; Table S1: Information for samples used in the present study; Table S2: Comparison of microbiota (ANOSIM) among different groups. CD, diet for cage-cultured fish; CG, gut of cage-cultured fish; CW, water from cage culture area; PD, diet for pond-cultured fish; PG, gut of pond-cultured fish; PS, sediment from pond; PW, water from pond; WG, gut of wild-caught fish; WW, water from natural sea area. *, significant association (*p* ≤ 0.05); **, significant association (*p* ≤ 0.01).

## References

[1] Gonzalez E.B., Umino T., Nagasawa K. (2008). Stock enhancement program for black sea bream, *Acanthopagrus schlegelii* (Bleeker), in Hiroshima Bay, Japan: A review. Aqua. Res..

[2] Tsuyuki A., Umino T. (2017). Spatial movement of black sea bream *Acanthopagrus schlegelii* around the oyster farming area in Hiroshima Bay, Japan. Fish. Sci..

[3] Yang H., Leng X., Du H., Luo J., Wu J., Wei Q. (2020). Adjusting the prerelease gut microbial community by diet training to improve the postrelease fitness of captive-bred *Acipenser dabryanus*. Front. Microbiol..

[4] Armstrong D.P., Seddon P.J. (2008). Directions in reintroduction biology. Trends Ecol. Evol..

[5] McFall-Ngai M., Hadfield M.G., Bosch T.C.G., Carey H.V., Domazet-Lošo T., Douglas A.E., Dubilier N., Eberl G., Fukami T., Gilbert S.F. (2013). Animals in a bacterial world, a new imperative for the life sciences. Proc. Natl. Acad. Sci. USA.

[6] Wang A.R., Ran C., Ringø E., Zhou Z.G. (2018). Progress in fish gastrointestinal microbiota research. Rev. Aquacult..

[7] Larsen A.M., Mohammed H.H., Arias C.R. (2014). Characterization of the gut microbiota of three commercially valuable warmwater fish species. J. Appl. Microbiol..

[8] Ghanbari M., Kneifel W., Domig K. (2015). A new view of the fish gut microbiome: Advances from next-generation sequencing. Aquaculture.

[9] Liu Y., Li X., Li J., Chen W. (2021). The gut microbiome composition and degradation enzymes activity of black Amur bream (*Megalobrama terminalis*) in response to breeding migratory behavior. Ecol. Evol..

[10] Liu H., Guo X., Gooneratne R., Lai R., Zeng C., Zhan F., Wang W. (2016). The gut microbiome and degradation enzyme activity of wild freshwater fishes influenced by their trophic levels. Sci. Rep..

[11] Liu Q., Lai Z., Gao Y., Wang C., Zeng Y., Liu E., Mai Y., Yang W., Li H. (2021). Connection between the gut microbiota of largemouth bass (*Micropterus salmoides*) and microbiota of the pond culture environment. Microorganisms.

[12] Mirghaed A.T., Yarahmadi P., Hosseinifar S.H., Tahmasebi D., Gheisvandi N., Ghaedi A. (2018). The effects singular or combined administration of fermentable fiber and probiotic on mucosal immune parameters, digestive enzyme activity, gut microbiota and growth performance of Caspian white fish (*Rutilus frisii kutum*) fingerlings. Fish Shellfish Immun..

[13] Scott K.P., Gratz S.W., Sheridan P.O., Flint H.J., Duncan S.H. (2013). The influence of diet on the gut microbiota. Pharmacol. Res..

[14] Dehler C.E., Secombes C.J., Martin S.A. (2017). Environmental and physiological factors shape the gut microbiota of Atlantic salmon parr (*Salmo salar* L.). Aquaculture.

[15] Egerton S., Culloty S., Whooley J., Stanton C., Ross R.P. (2018). The gut microbiota of marine fish. Front. Microbiol..

[16] Kashinskaya E.N., Simonov E.P., Kabilov M.R., Izvekova G.I., Andree K.B., Solovyev M.M. (2018). Diet and other environmental factors shape the bacterial communities of fish gut in an eutrophic lake. J. Appl. Microbiol..

[17] Huang X.F. (2000). Survey, Observation and Analysis of Lake Ecology.

[18] Sun Z., Liu W., Bao Q., Zhang J., Hou Q., Kwok L., Zhang H. (2014). Investigation of bacterial and fungal diversity in tarag using high-throughput sequencing. J. Dairy Sci..

[19] Callahan B.J., Mcmurdie P.J., Rosen M.J., Han A.W., Johnson A.J., Holmes S.P. (2016). DADA2: High-resolution sample inference from Illumina amplicon data. Nat. Methods.

[20] Quast C., Pruesse E., Yilmaz P., Gerken J., Schweer T., Yarza P., Peplies J., Gloeckner F.O. (2013). The SILVA ribosomal RNA gene database project: Improved data processing and web-based tools. Nucleic Acids Res..

[21] Bokulich N.A., Kaehler B.D., Rideout J.R., Dillon M., Bolyen E., Knight R., Huttley G.A., Caporaso J.G. (2018). Optimizing taxonomic classification of marker-gene amplicon sequences with QIIME 2′s q2-feature-classifier plugin. Microbiome.

[22] Dixon P. (2003). VEGAN, a package of R functions for community ecology. J. Veg. Sci..

[23] Legendre P., Legendre L. (1998). Numerical Ecology (2nd English Edition), Developments in Environmental Modelling 20.

[24] Warton D.I., Wright S.T., Wang Y. (2012). Distance-based multivariate analyses confound location and dispersion effects. Methods Ecol. Evol..

[25] Knights D., Kuczynski J., Charlson E.S., Zaneveld J., Mozer M.C., Collman R.G., Bushman F.D., Knights R., Kelley S.T. (2011). Bayesian community-wide culture-independent microbial source tracking. Nat. Methods.

[26] Langille M.G.I., Zaneveld J., Caporaso J.G., McDonald D., Knights D., Reyes J.A., Clemente J.C., Burkepile D.E., Vega Thurber R.L., Knight R. (2013). Predictive functional profiling of microbial communities using 16S rRNA marker gene sequences. Nat. Biotech..

[27] Rombout J.H., Abelli L., Picchietti S., Scapigliati G., Kiron V. (2011). Teleost intestinal immunology. Fish Shellfish. Immunol..

[28] Desai A.R., Links M.G., Collins S.A., Mansfield G.S., Drew M.D., Van Kessel A.G., Hill J.E. (2012). Effects of plant-based diets on the distal gut microbiome of rainbow trout (*Oncorhynchus mykiss*). Aquaculture.

[29] Givens C., Ransom B., Bano N., Hollibaugh J. (2015). Comparison of the gut microbiomes of 12 bony fish and 3 shark species. Mar. Ecol. Prog. Ser..

[30] Parma L., Pelusio N.F., Gisbert E., Esteban M.A., D’Amico F., Soverini M., Canadela M., Dondi F., Gatta P.P., Bonaldo A. (2020). Effects of rearing density on growth, digestive conditions, welfare indicators and gut bacterial community of gilthead sea bream (*Sparus aurata*, L. 1758) fed different fishmeal and fish oil dietary levels. Aquaculture.

[31] Zhao R., Symonds J.E., Walker S.P., Steiner K., Nowak B.F. (2020). Salinity and fish age affect the gut microbiota of farmed chinook salmon (*Oncorhynchus tshawytscha*). Aquaculture.

[32] Huang Q., Sham R.C., Deng Y., Mao Y., Wang C., Zhang T., Leung K.M.Y. (2020). Diversity of gut microbiomes in marine fishes is shaped by host-related factors. Mol. Ecol..

[33] Deng H.W., Liu F., Li G.H., Bao W.Y., Zou S.B., Zheng P.F., Gong J., Sun R.J. (2019). Response of gut bacterial community in black sea bream to different feather meal in feed. Oceanol. Limnol. Sin..

[34] Roeselers G., Mittge E., Stephens W., Parichy D., Cavanaugh C., Guillemin K., Rawls J. (2011). Evidence for a core gut microbiota in the zebrafish. ISME J..

[35] Smriga S., Sandin S.A., Azam F. (2010). Abundance, diversity, and activity of microbial assemblages associated with coral reef fish guts and feces. FEMS Microbiol. Ecol..

[36] Kuang T., He A., Lin Y., Huang X., Liu L., Zhou L. (2020). Comparative analysis of microbial communities associated with the gill, gut and habitat of two filter-feeding fish. Aquacul. Rep..

[37] Li T., Long M., Gatesoupe F.J., Zhang Q., Li A., Gong X. (2015). Comparative analysis of the intestinal bacterial communities in different species of carp by pyrosequencing. Microb. Ecol..

[38] Semova I., Carten J.D., Stombaugh J., Mackey L.C., Knight R., Farber S.A., Rawls J.F. (2012). Microbiota regulate intestinal absorption and metabolism of fatty acids in the zebrafish. Cell Host Microbe.

[39] Sun P., Ling J.Z., Zhang H., Tang B.J., Jiang Y.Z. (2021). Diet composition and feeding habits of black sea bream (*Acanthopagrus schlegelii*) in Xiangshan Bay based on high-throughput sequencing. Acta Ecol. Sin..

[40] Li J., Ni J., Li J., Wang C., Li X., Wu S., Zhang T., Yu Y., Yan Q. (2014). Comparative study on gastrointestinal microbiota of eight fish species with different feeding habits. J. Appl. Microbiol..

[41] Wu S.G., Tian J.Y., Gatesoupe F.J., Li W.X., Zou H., Yang B.J., Wang G.T. (2013). Intestinal microbiota of gibel carp (*Carassius auratus gibelio*) and its origin as revealed by 454 pyrosequencing. World J. Microbiol. Biotechnol..

[42] Vadstein O., Attramadal K.J.K., Bakke I., Forberg T., Olsen Y., Verdegem M., Giatsis C., Skjermo J., Aasen I.M., Gatesoupe F.J. (2018). Managing the Microbial Community of Marine Fish Larvae: A Holistic Perspective for Larviculture. Front. Microbiol..

[43] Wu S., Wang G., Angert E., Wang W., Li W., Zou H. (2012). Composition, diversity, and origin of the bacterial community in Grass carp intestine. PLoS ONE.

