# Supplementary material for: Relationships between the Gut Microbiota of Juvenile Black Sea Bream (Acanthopagrus schlegelii) and Associated Environment Compartments in Different Habitats
Source: Microorganisms. 2021 Dec 10;9(12):2557. doi: 10.3390/microorganisms9122557 (PMC8705249; doi:10.3390/microorganisms9122557)
Supplement: Supplementary file 1 [file microorganisms-09-02557-s001.zip › 1 Supplymentary Tables.pdf]

Table S1 Information for samples used in the present study.

Group	Habitat	Sample	Number
WG	natural sea area	gut content	9
WW		water	5
CG	cage culture area	gut content	9
CW		water	5
CD		diet	3
PG	pond culture area	gut content	9
PW		water	3
PD		diet	3
PS		sediment	3

Table S2 Comparison of microbiota (ANOSIM) among different groups.

Group1	Group2	Sample size	Permutations	*R*	*p*-value	*q*-value
all	-	49	999	0.820	0.001**	-
CD	PD	6	999	1	0.099	0.108
CD	PS	6	999	1	0.104	0.108
CD	PW	6	999	1	0.108	0.108
CD	WG	12	999	0.734	0.005**	0.012
CD	CG	12	999	0.749	0.005**	0.012
CD	PG	12	999	0.550	0.005**	0.013
CD	WW	8	999	1	0.020*	0.028
CD	CW	8	999	1	0.024*	0.030
PD	PS	6	999	1	0.106	0.108
PD	PW	6	999	1	0.097	0.108
PD	WG	12	999	1	0.003**	0.010
PD	CG	12	999	1	0.006**	0.013
PD	PG	12	999	0.776	0.003**	0.010
PD	WW	8	999	1	0.016*	0.025
PD	CW	8	999	1	0.022*	0.029
PS	PW	6	999	1	0.098	0.108
PS	WG	12	999	1	0.005**	0.012
PS	CG	12	999	1	0.007**	0.013
PS	PG	12	999	1	0.007**	0.013

PS	WW	8	999	1	0.027*	0.032
PS	CW	8	999	1	0.017*	0.026
PW	WG	12	999	1	0.005**	0.012
PW	CG	12	999	1	0.007**	0.013
PW	PG	12	999	0.962	0.009**	0.015
PW	WW	8	999	1	0.023*	0.030
PW	CW	8	999	1	0.018*	0.026
WG	CG	18	999	0.330	0.001**	0.005
WG	PG	18	999	0.488	0.001**	0.005
WG	WW	14	999	1	0.001**	0.005
WG	CW	14	999	0.990	0.001**	0.005
CG	PG	18	999	0.512	0.001**	0.005
CG	WW	14	999	1	0.001**	0.005
CG	CW	14	999	1	0.001**	0.005
PG	WW	14	999	0.993	0.003**	0.010
PG	CW	14	999	1	0.002**	0.009
WW	CW	10	999	0.992	0.008*	0.014

CD: diet for cage-cultured fish; CG: gut of cage-cultured fish; CW: water from cage culture area; PD: diet for pond-cultured fish; PG: gut of pond-cultured fish; PS: sediment from pond; PW: water from pond; WG: gut of wild-caught fish; WW: water from natural sea area. Note: *: Significant association ($P \leq 0.05$); **: Significant association ($P \leq 0.01$).
