# Supplementary figures and images for: Relationships between the Gut Microbiota of Juvenile Black Sea Bream (Acanthopagrus schlegelii) and Associated Environment Compartments in Different Habitats
Source: Microorganisms. 2021 Dec 10;9(12):2557. doi: 10.3390/microorganisms9122557 (PMC8705249; doi:10.3390/microorganisms9122557)

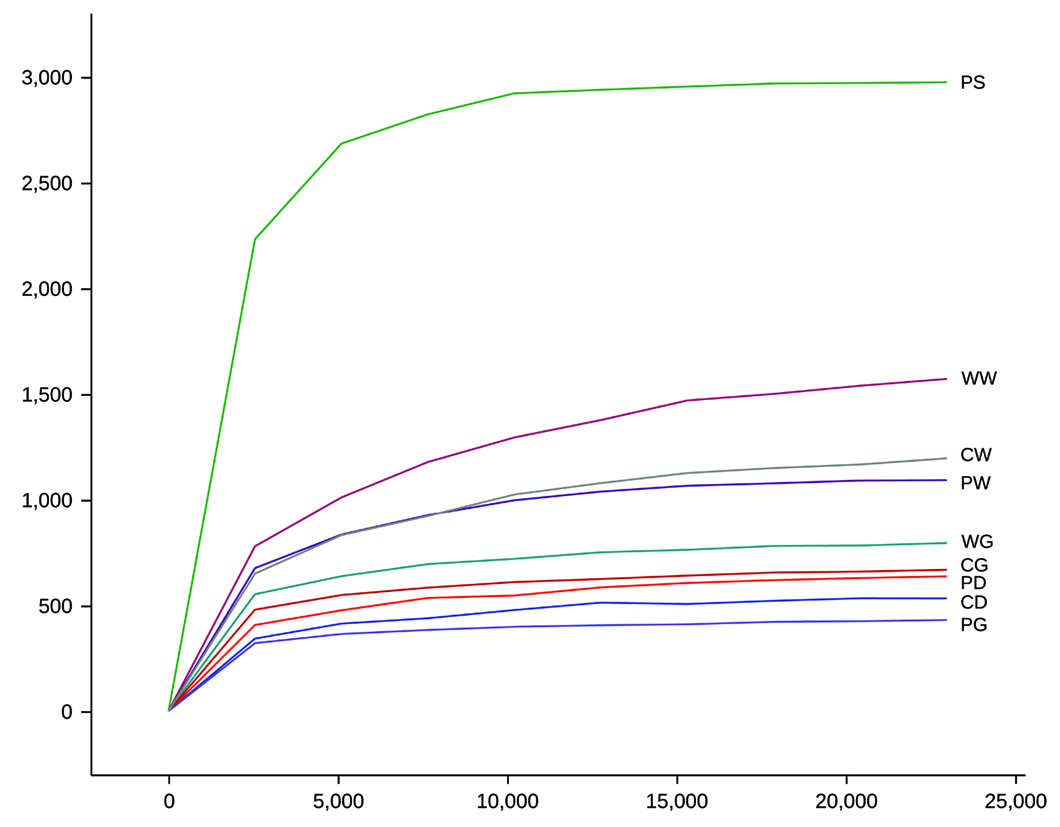

Supplement: Supplementary file 1 [file microorganisms-09-02557-s001.zip › Fig.S1.tif]

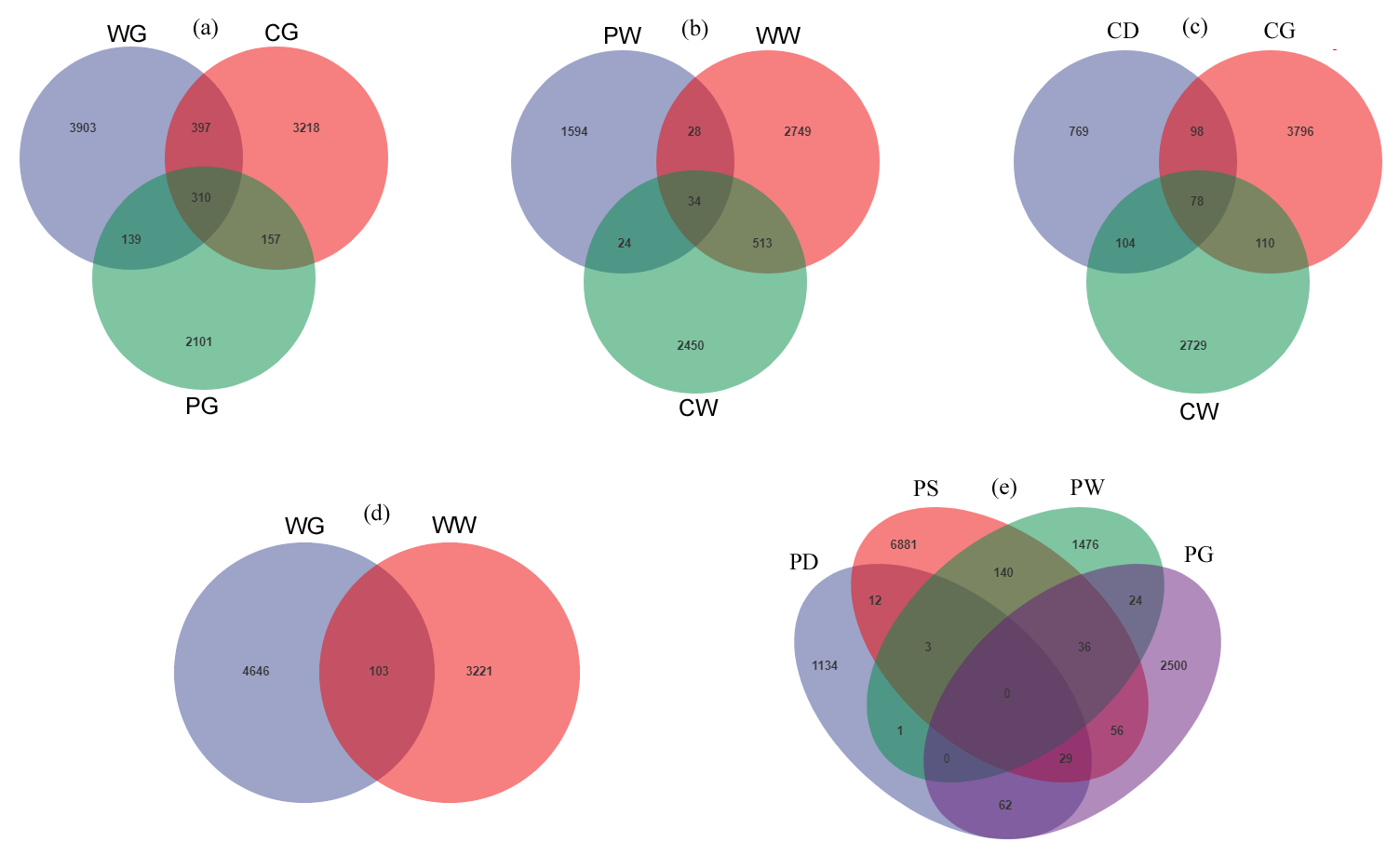

Supplement: Supplementary file 1 [file microorganisms-09-02557-s001.zip › Fig.S2.tif]

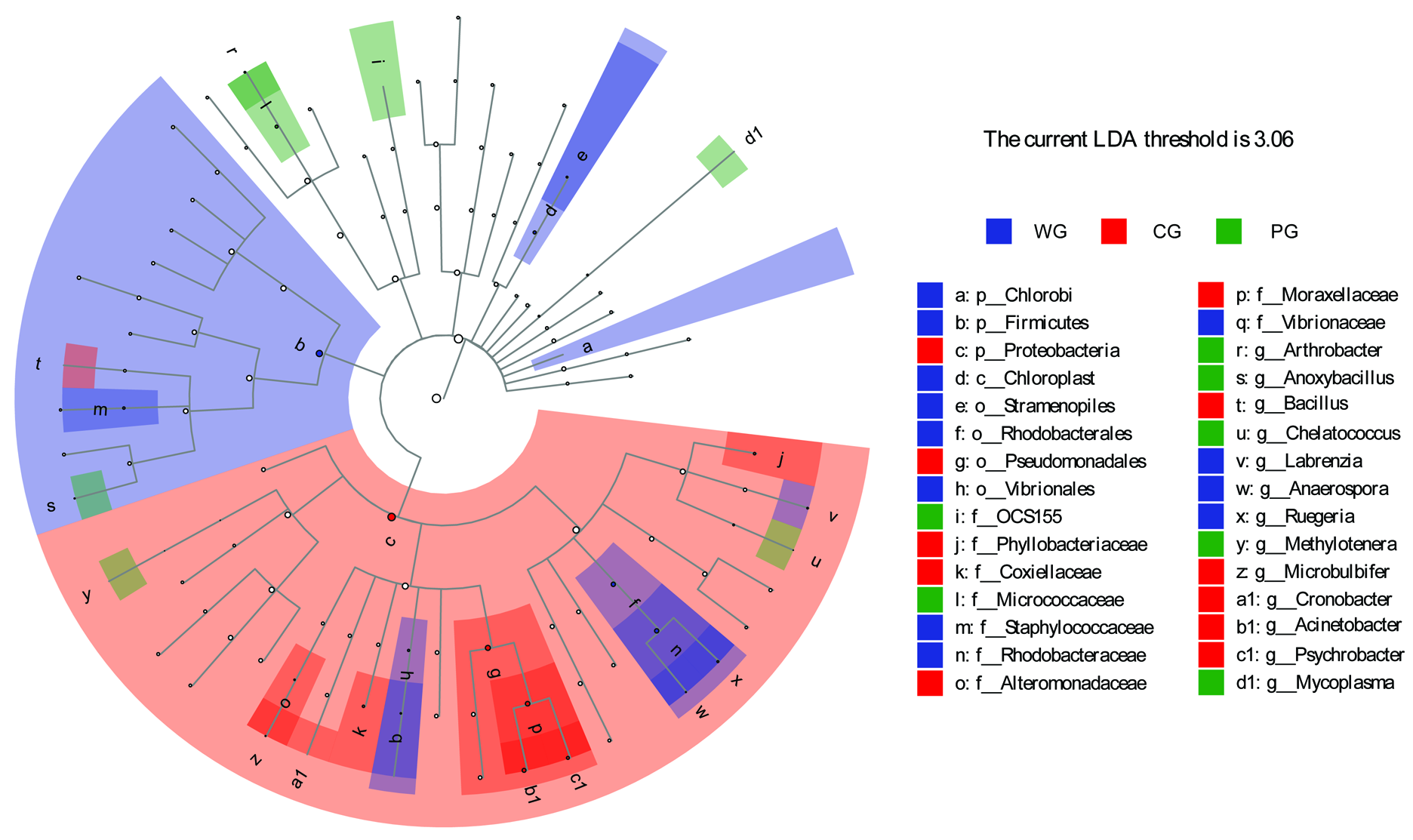

Supplement: Supplementary file 1 [file microorganisms-09-02557-s001.zip › Fig.S4.tif]
